# Abdominal Aortic Occlusion by Hydatid Cysts

**DOI:** 10.1055/s-0039-3401994

**Published:** 2020-06-29

**Authors:** Uma Debi, Vikas Bhatia, M.S. Sandhu

**Affiliations:** 1 Department of Radiodiagnosis and Imaging, Postgraduate Institute of Medical Education and Research, Chandigarh, Chandigarh, India

**Keywords:** hydatid, aorta, albendazole, MRI

## Abstract

Hydatid disease is a parasitic infestation caused by the larval stage of *Echinococcus* . It can infest any part of the body; however, aortic hydatid disease is rare. Involvement of the abdominal aorta is usually due to embolization from cardiac hydatid cysts or direct invasion and can be present at intravascular or intramural locations. Aortic hydatid disease may present with fatal complications, such as anaphylaxis, pseudoaneurysm formation, systemic embolism, and arterial occlusion.

A 36-year-old woman presented with history of dull aching pain in the abdomen, on and off fever, and intermittent cough for 1 month. She had been operated for frontal lobe hydatid cyst 6 months earlier. Her postoperative status was uneventful, and she was maintained on albendazole therapy.

On physical examination, splenomegaly was found with weak femoral pulses. Her biochemical and hematological findings were unremarkable except for elevated erythrocyte sedimentation rate. Chest radiography and electrocardiogram were normal.

Biphasic computed tomography revealed multiple hydatid cysts, with the largest measuring 3 cm × 2.4 cm in the abdominal aorta. Volume rendered images ( Fig. 1A ) revealed complete occlusion of the infrarenal aorta with abrupt cut-off in its infrarenal segment at the L2 level with partial reformation of distal bilateral common iliac artery by multiple collaterals. Mesenteric collaterals, such as the marginal artery of Drummond and the arc of Riolan were highly developed. Dilated, tortuous intercostals, and internal mammary arteries were also seen. Sagittal magnetic resonance imaging using True fast technology with steady state precession (TrueFISP) sequence shows multiple hydatid cysts in the infrarenal aorta ( Fig 1B ).

**Fig. 1:**
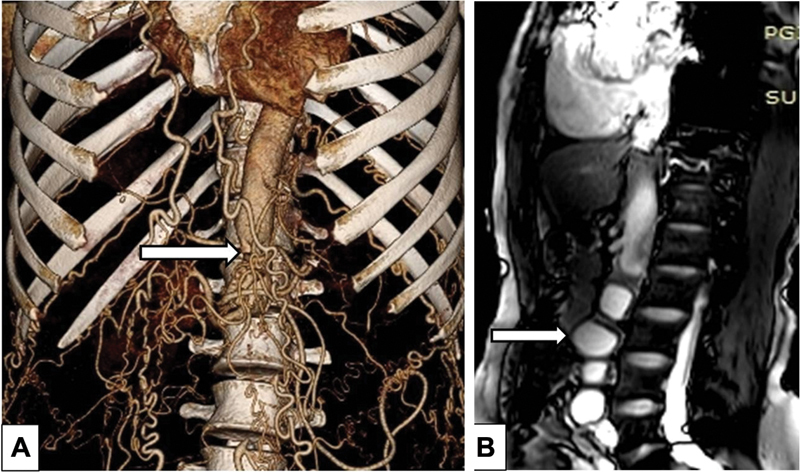
( **A** ) CT scan demonstrating complete aortic occlusion (arrow) and highly developed collaterals (via internal mammory and abdomen vessels), see text. ( **B** ) Hydatid cysts filling and occluding the infrarenal aorta. The largest one (2.4 cm × 3 cm) is marked by the arrow.

Hydatid disease is a parasitic infestation caused by the larval stage of *Echinococcus* . Hydatid disease can infest any part of the body; however, arterial involvement is rare. 1 2 Involvement of the abdominal aorta is usually due to embolization from cardiac hydatid cysts or direct invasion and can be present at intravascular or intramural locations. 3

Aortic hydatid disease can present with fatal complications, such as anaphylaxis, pseudoaneurysm formation, systemic embolism, and arterial occlusion. Resection with graft interposition has been described successfully in few case reports. Systemic medical therapy with albendazole along with surgery can help in eradication and prevention of recurrence.
